# Data enabled prediction analysis assigns folate/biopterin transporter (BT1) family to 36 hypothetical membrane proteins in Leishmania donovani

**DOI:** 10.6026/97320630015697

**Published:** 2019-10-18

**Authors:** Nithin Ravooru, Ojal Sarah Paul, Holenarasipur Gundurao Nagendra, Nitish Sathyanarayanan

**Affiliations:** 1 Department of Biotechnology, Sir M Visvesvaraya Institute of Technology,Bangalore; 2 National Centre for Biological Sciences, Tata Institute of Fundamental Research,Bangalore; 3 University of Pittsburgh Medical Center, Pittsburgh, Pennsylvania

**Keywords:** Leishmaniadonovani, hypothetical protein, membrane proteins, MFS, Folate/biopterin transporters

## Abstract

Leishmaniasis is a neglected tropical disease caused by the pathogenic protozoan Leishmania donovani and it is transmitted by an infected sand fly. Approximately 0.4 million cases of Visceral Leishmaniasis are reported across the globe every year, of which 67% is from the Indian subcontinent. The currently available drugs have not been effective owing to their high toxicity levels, inadequate specificity, drug resistance, extended treatment periods and/or prohibitive prices. For this reason, hypothetical proteins in this pathogen, which constitute about 67% of its proteome, must be distinctly characterized and studied for their potential role as drug targets for Leishmaniasis. Domain information from PFAM and functional information from GO has been used to assign putative functions to 36 hypothetical membrane proteins in this protozoan. Furthermore, as a case study, we have performed a thorough sequence level characterization of a hypothetical protein E9BPD7 from the BT1 family of membrane proteins that transports folate/biopterin. Phylogenetic analyses of E9BPD7 have revealed interesting evolutionary correlations to BT1 family and MFS superfamily, which have significant roles in a number of diseases and drug resistance pathways.

## Background

Leishmaniasis is a neglected vector-borne disease caused by protozoan flagellates and transmitted by phlebotomine sand flies. The different clinical manifestations of Leishmaniasis include Cutaneous Leishmaniasis (CL), Mucocutaneous Leishmaniasis (MCL), Visceral Leishmaniasis (VL) and Post Kala Azar Dermal Leishmaniasis (PKDL) [1].VL is popularly known as Kala-Azar in the Indian Subcontinent and is mainly caused by the protozoan Leishmania donovani.Amongst the four manifestations of Leishmaniasis, VL is fatal and its major clinical symptoms include prolonged fever, anaemia, splenomegaly, prominent wasting, death from organ failure and opportunistic infections when left untreated [2].According to the studies carried out by the WHO Leishmaniasis Control Team, the annual incidence ranges of VL and CL is approximately 0.2-0.4 million and 0.8-1.2 million cases respectively (as estimated by country and epidemiological region) [3].

Hypothetical proteins define the unknown elements of the proteomes in biological systems. In several pathogenic systems, the analysis of hypothetical proteins has provided insights to the molecular function of these proteins and led to potential therapeutic targets [4- 5]. The growing need for identification of newer drug targets can thus be addressed by considering the hypothetical proteome of the etiological agent Leishmania donovani. Membrane proteins form the connecting interface between the intracellular and the extracellular environments. They are also involved in mediating the exchange of molecules and cell communication. Additionally, there is little information about the nature of these bio molecules and their role in communication with extracellular environment. Thus, identification of cell surface proteins and transmembrane proteins would help in determining crucial systems that could be explored as potential targets [6-7].Several in silico studies on membrane proteins have helped understanding the role of this class of proteins. Using computational approaches, 7 novel outer membrane proteins and other non-paralogous proteins were identified in Edwardsiella tarda, a fish pathogen that infects Daniorerio (zebra fish) [8].In another study, the hypothetical membrane proteins in Trypanosoma cruziwere characterised and 54 proteins involved in signal transduction processes were meticulously investigated following the identification using computer aided computer models [6]. Also, an in silico analysis of the Neisseria meningitidessero-group B proteome revealed 9 outer membrane proteins, among other potential drug targets, that could be potential vaccine candidates towards treatment of Meningococcal disease [9].

Furthermore, computational annotation of hypothetical proteins has been extensively carried out in Mycobacterium tuberculosis (Mtb) using homology information from databases like COG and GO [10]. Homology based fold predictions have also been widely used approaches to computationally annotate and attribute functional domain information to proteins. Functional domains were assigned to 64 proteins using homology and fold prediction approaches in Mycobacterium tuberculosis [11]. Similarly, using various approaches like homology, structure and fold prediction; 219 structural folds were computationally annotated to hypothetical proteins within M. tb [12]. Hence, a comprehensive in silico analysis of the hypothetical membrane proteins of Leishmania donovani was carried out to understand their roles in the biology of this notorious protozoan.

## Methodology

### Databases employed:

The hypothetical sequences belonging to Leishmaniadonovani were retrieved from UNIPROT (Release 2014_02)[13]. The annotated Swissprot database (March 2014) and the Human Proteome database (August_2014) were used to perform standalone BLAST (version 2.2.29+) searches. Also, Pfam A (version 27.0) domain database was used for HMMER (version 3.1b1) searches to assign domain information [14]. COG database was utilized to find orthologous group information for the membrane proteins in this study[15].

### Tools for functional analysis:

HMMTOP[16] and TMHMM were used to predict membrane proteins from hypothetical dataset[17]. The standalone version of HMMER (version 3.1b1) was used to assign domain information to the sequences whenever necessary. Standalone version of BLAST program was used to identify homologs for our hypothetical sequences[18].Blast2GO was employed to associate functional information using GO terms[19].The CD-Hit tool was used to cluster the sequences at the required identity cut-off[20]. Phylogenetic analysis of protein sequences, was carried using MEGA 5.0 andRaXML with both Maximum Likelihood (ML) and Neighbour Joining (NJ) methods[21-22].The alignment was performed using Muscle for 10 iterations[23]. JTT model was used for phylogeny, which has been previously used for performing phylogeny of membrane proteins as it is known to maximize the alignment of helices interspersed with long gaps[24]. The resulting trees were visualized using Figtree (version 1.4).

## Results

**Figure 1:**
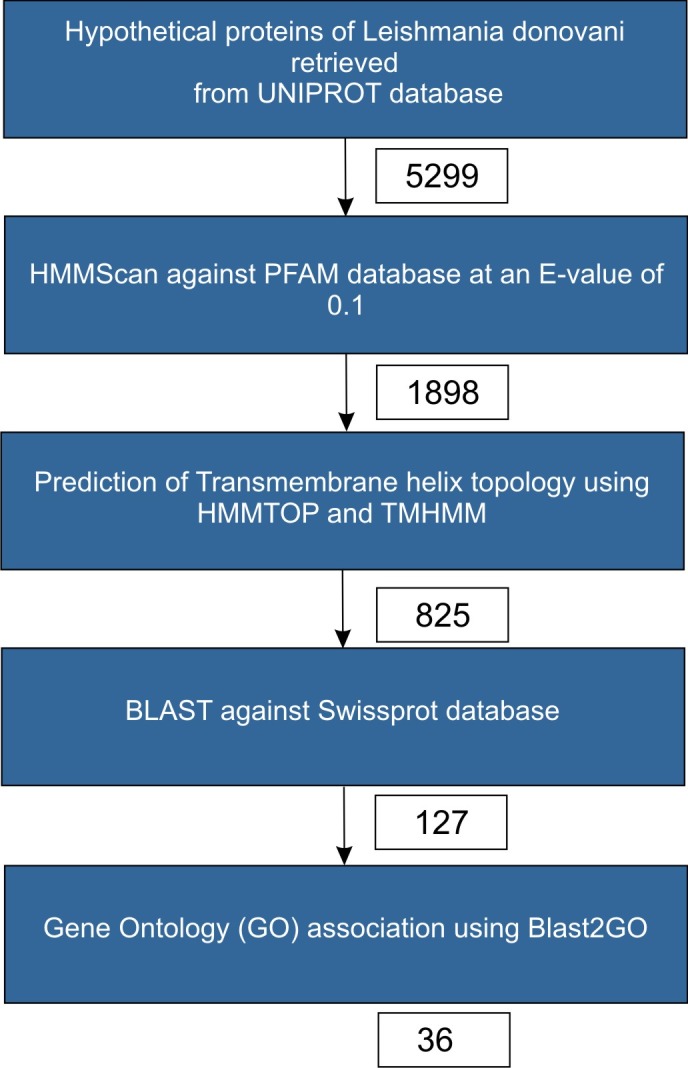
Protocol used in sequence processing

Figure 1 shows the flowchart depicting the methodology adopted in this study. 5,299 sequences belonging to Leishmaniadonovani (strain BPK282A1) andtermed as 'uncharacterised' were retrieved from UNIPROT database. Further, HMM scan was performed against the PFAM database (version 27.0) resulting in 1,898 sequences finding a domain association at an E value of 10-3 with domain coverage of more than 50%. Also, the combined prediction of HMMTOP and TMHMM resulted in 825 sequences being predicted to have putative membrane protein topology[16-17].These 825 sequences were searched for possible homologs using BLAST against the Swissprot database at an E-value of 10-5 (to avoid any false positives)[25]. Sequence identity cut-off of 30% and a coverage cut-off of 70% were used as primary criteria to select true positives, which resulted in 127 sequences finding homologs in the Swissprot database. GO mapping using Blast2GO tool was performed for these 127 sequences which resulted in 36 sequences having a membrane term in the Cellular component of the GO annotation. Thus, 36 uncharacterized proteins, which were predicted to have membrane topology using HMMTOP and TMHMM, were associated to a PFAM domain covering more than 50% of the query's length and assigned with a GO term using Blast2GO tool.

**Figure 2:**
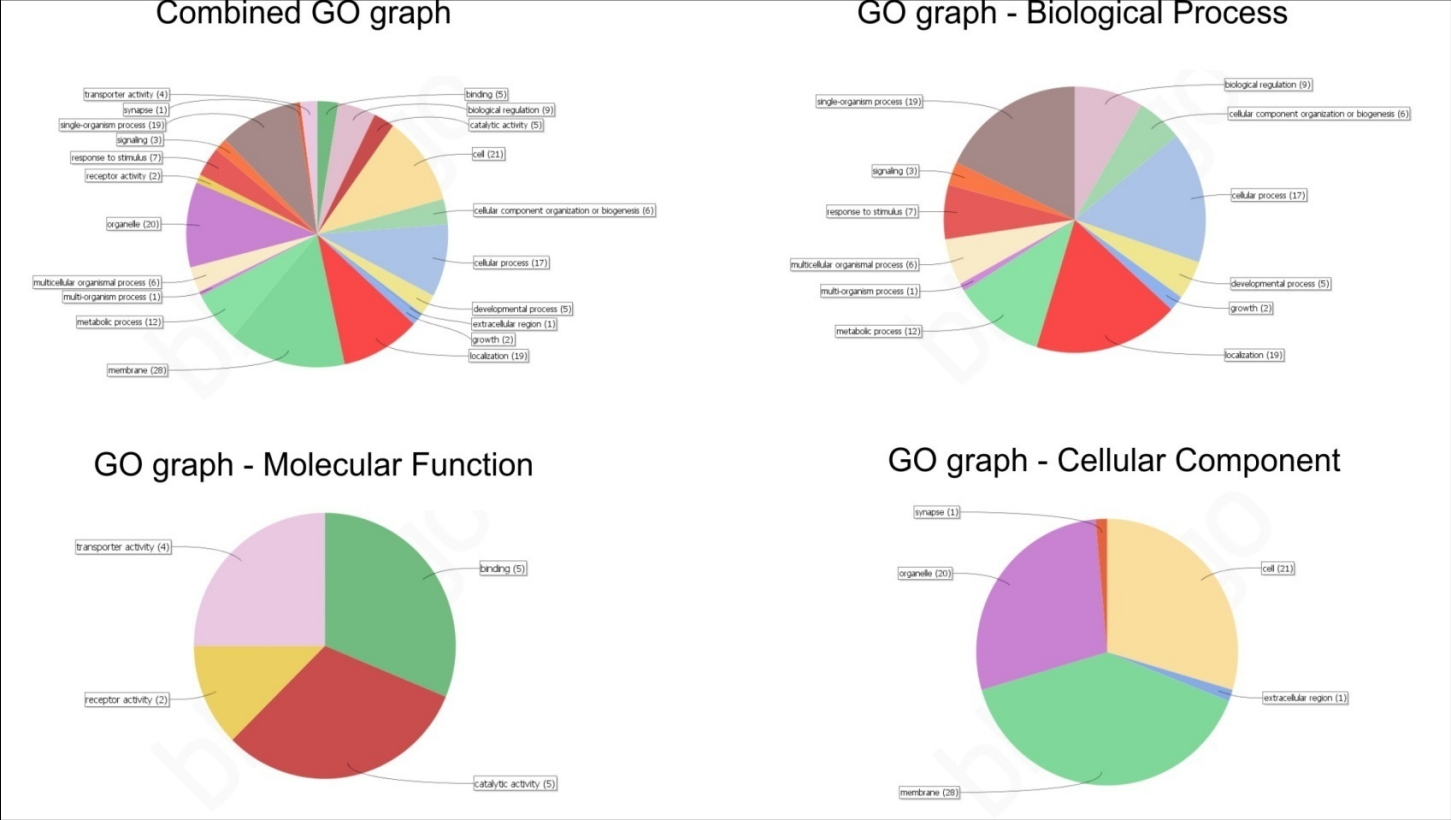
(A) Combined GO graph; (B) Distribution of biological process of the 36 sequences. (C) Distribution of molecular function the 36 sequences; (D) Distribution of cellular component of the 36 sequences

In the present study, we have utilized the data repository in publicdatabases and bioinformatics tools to assign putative functions to the hypothetical membrane proteins in Leishmaniadonovani. Amongst the 7,960 sequences that make up the proteome 5,299 proteins in this protozoan are termed uncharacterized/hypothetical. This amounts to a monumental figure of 65% of the proteome that is not associated with any functional information. As detailed in Figure 1 and exercises thereof, 36 sequences have been characterized with domain information from PFAM and functional annotation was given from GO. All the information related to GO terms, Inter proscan domain associations, homologs identified during the BLAST step of Blast2GO analysis for these 36 sequences are presented in Table 1. In the first step of Blast2Go, a BLAST search is performed to identify sequence homologs to our query. These sequence homologs are considered for attributing GO terms to the query in further steps. The sequence similarity distribution amongst the BLAST hits obtained in the first step of Blast2Go analysis indicated no BLAST hits were present with less than 30% sequence similarity with respect to the query (data not shown). Also, only hits with significant E-values were considered for further analysis (data not shown). Figure 2A–Drepresents the combined graphs depicting the biological processes, molecular functions and cellular components of these 36 sequences respectively. As may be appreciated from Figure 2 A-D, a sequence may have association to more than one GO term.Further, these 36 sequences were associated to a COG family using STRING-DB search. 35 of the 36 sequences found a COG family while E9BPD7 did not find any COG family. Therefore, a detailed sequence characterization of E9BPD7 was taken up as a case study.

## Case study 1: Analysis of E9BPD7 belonging to the BT1 family and MFS super family

Sequence and Phylogenetic characterization of E9BPD7 (TritrypDB accession: LdBPK_323870) was performed, as this protein does not belong to any COG family. However, upon HMMscan against PFAM database, the protein was interestingly associated with BT1 family (PF03092.11) and shared a similarity (>30%) with the folate transporter of Arabidopsis thaliana. The BT1 family of transmembrane proteins is a member of clan - major facilitator superfamily (MFS). MFS is a predominant family of membrane transporters present in bacteria, archaea and eukarya. Based on sequence signatures and with the help of phylogeny, Pao et al.(1998) have previously classified MFS into 17 unique families[26].However, in the classification suggested elsewhere [28], BT1 has not been classified as a separate family. Hence, phylogenetic analysis was performed to understand the evolutionary relationship of BT1 family with other members of MFS superfamily and to understand the phyletic distribution of various members of BT1 family.

**Figure 3:**
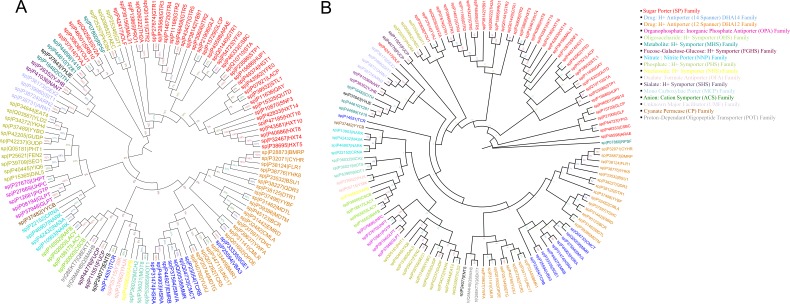
(A) Tree 1A: Phylogenetic Clustering of 17 MFS member families defined by Pao et al. (1998) [26] obtained using MEGA 5.0, Maximum Likelihood method with 100 bootstrap replications. (B) Tree 1B: Phylogenetic Clustering of 17 MFS member families defined by Pao et al. (1998) [26] obtained using RaXML, Maximum Likelihood method with 500 bootstrap replications.

We re-constructed the phylogenetic tree described in [28] using Maximum likelihood (ML) method with 2 different tools. 176 valid sequences belonging to 17 subfamilies were used for building the phylogenetic tree. Clustering of 176 sequences was performed at an identity cut-off of 70%, which reduced the number of representative sequences to 121 from a total of 176. The resulting tree (Tree 1) containing 17-member families of MFS are shown in Figure 3. MEGA tool was used to build ML tree with 100 bootstrap replications (Figure 3A) while RaXML was used to build the tree with 500 bootstrap replications (Figure 3B). Additionally, a phylogenetic tree was also built using Neighbour Joining method with 500 bootstrap replications (data not shown) to showequivalency of the phylogenetic clustering using different methods. The tree described by Pao et al.was built using the TREE program [27].Since, BT1 family has not been classified as a separate family in the previous tree by Paoet al. we performed clustering of BT1 family along with the 17 well defined member families of MFS (121 sequences used in Figure 3).There are a total of 726 sequences distributed over 173 species in BT1 family in PFAM (version 27.0).

**Figure 4:**
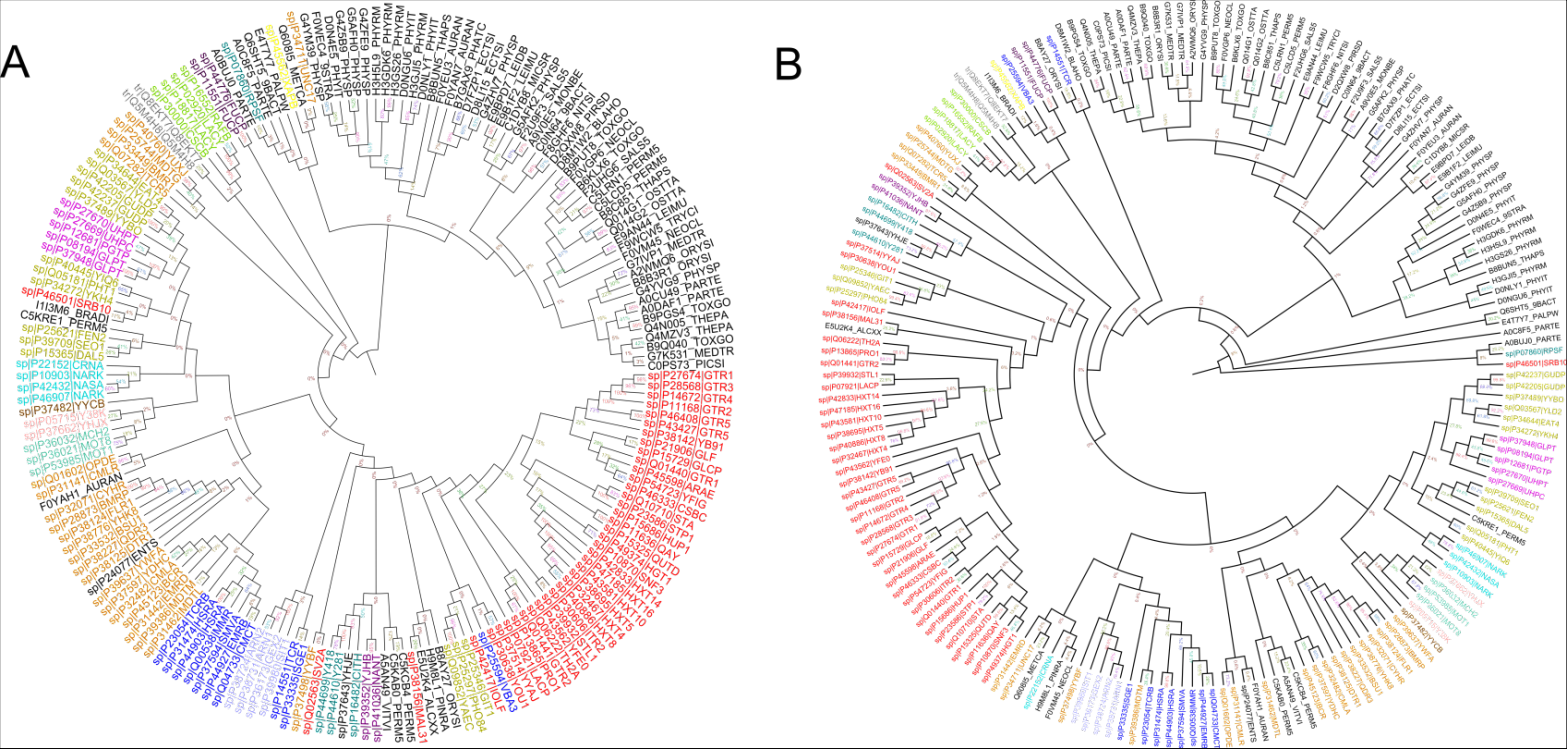
(A) Tree 2A: Phylogenetic clustering of BT1 family members with the 17 MFS family members defined by Pao et al. (1998) [26], obtained using MEGA 5.0, Maximum Likelihood method with 100 bootstrap replications. (B): Tree 2B: Phylogenetic clustering of BT1 family members with the 17 MFS family members defined by Pao et al. (1998) [26] obtained using RaXML, Maximum Likelihood method with 500 bootstrap replications.

In order to demonstrate BT1 as a separate functional family within MFS, a phylogenetic tree (Tree 2) was constructed with 121 representative sequences of 17 member families (as for Tree1 in Figure 3A) along with the 67 representative sequences belonging to BT1 family. Like Tree 1, the phylogenetic tree was constructed with 2 different programs with 2 different bootstrap replications. MEGA tool was used to build ML tree with 100 bootstrap replications (Figure 4A) while RaXML was used to build the tree with 500 bootstrap replications (Figure 4B). The resulting tree is depicted as Figure 4.Furthermore, phylogenetic clustering of the members of BT1 family alone was performed to understand the sequence level clustering within this class of proteins. The special interest in clustering of BT1-family members from Trypanosomatids motivated us to perform a phylogenetic analysis of the sequences.

To retain only representative sequences from this family, we clustered 726 sequences at 35% sequence identity cut-off which resulted in obtaining 67 representative sequences for BT1 family.

**Figure 5:**
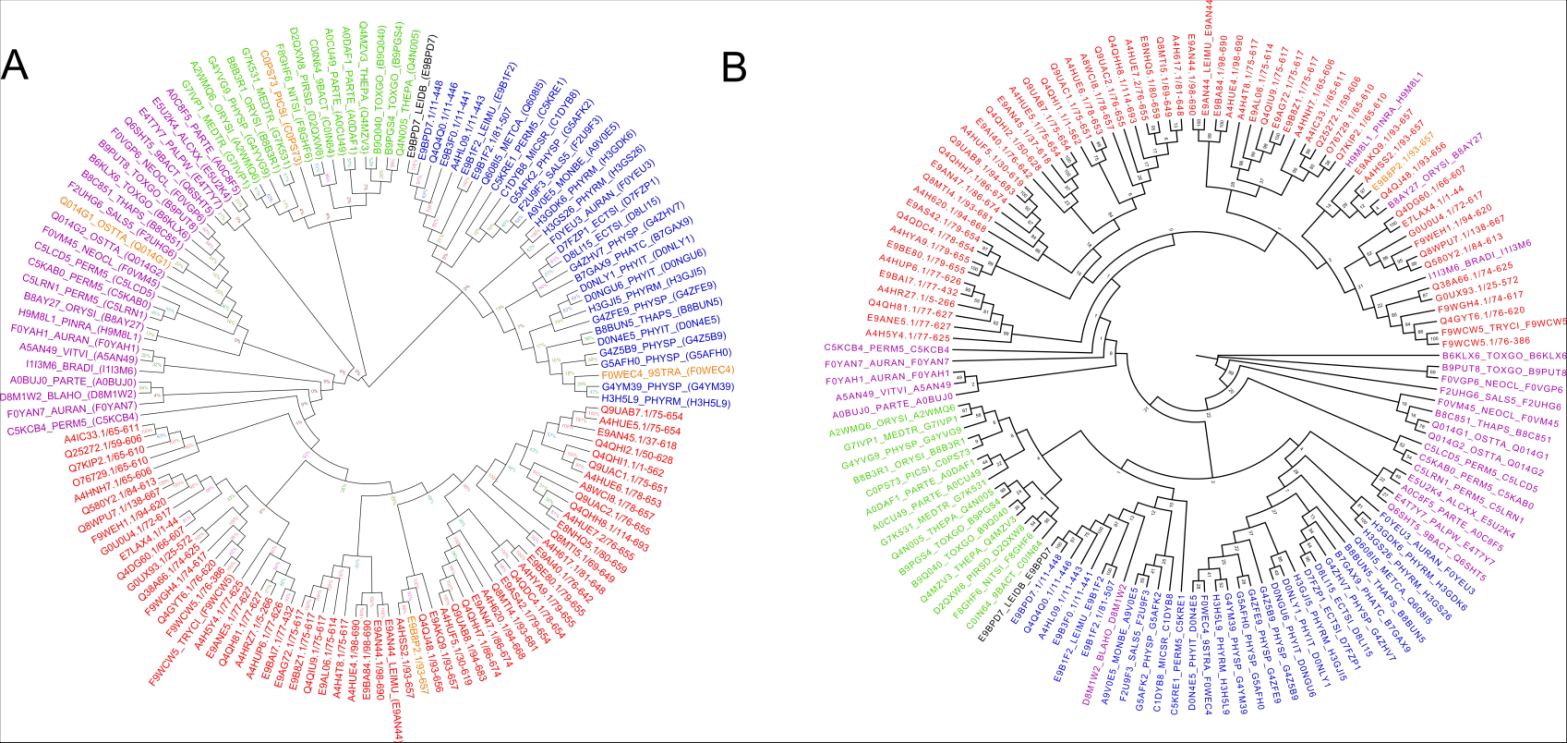
(A) Tree 3A: Phylogenetic clustering of BT1 family members, obtained using MEGA 5.0, Maximum Likelihood method with 100 bootstrap replications. (B) Tree 3B: Phylogenetic clustering of BT1 family members, obtained using RaXML, Maximum Likelihood method with 500 bootstrap replications.

**Figure 6:**
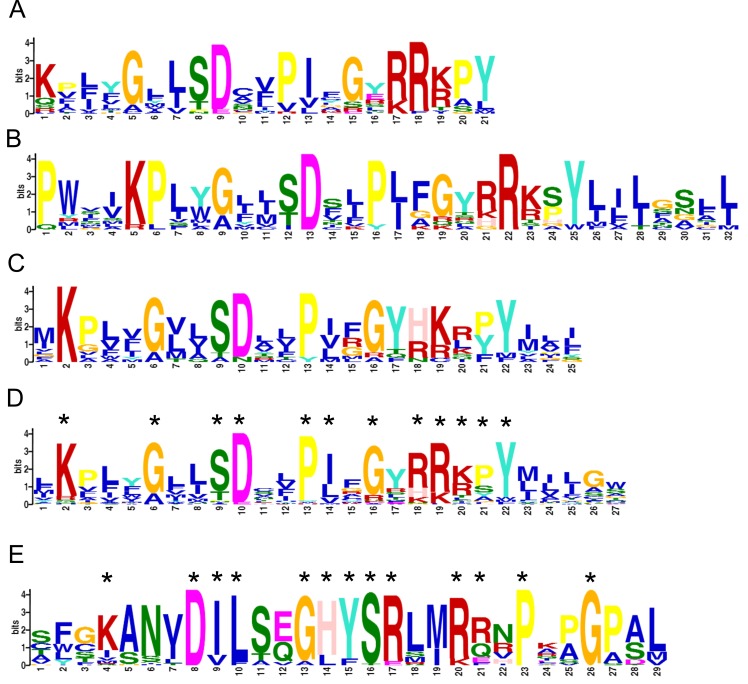
(A) MEME Motif for Blue Cluster; (B) MEME Motif for Green Cluster; (C) MEME Motif for Purple Cluster; (D) Combined MEME Motif for the Blue, Green and Purple Cluster; (E) Unique MEME Motif for Red Cluster

For this reason, we retrieved all of the 66 sequences from BT1 family belonging to Trypanosomatids which upon CD-hit clustering of 100%, reduced to 64 protein sequences. A phylogenetic tree was built using these 64 sequences from Trypanosomatids and 67 representative sequences belonging to other species of BT1 family (as used in Tree 2, Figure 4) along with our query of interest, E9BPD7. Two independent methods were used for building the phylogenetic tree. MEGA tool was used to build ML tree with 100 bootstrap replications (Figure 5A) while RaXML was used to build the tree with 500 bootstrap replications (Figure 5B).Using the MEME suite, we identified class specific motifs for the 4 different clusters within the tree (Figure 5B) [28].Figure 6 shows the MEME output/sequence motifs for the four clusters.

## Discussion

BT1 family is part of Multi Facilitator Superfamily (MFS), a large class of membrane proteins involved in the transport of various molecules across the membrane. MFS is one of the two largest superfamilies of membrane transport proteins that is virtually distributed among all the recognised organism phyla[26]. As of 2012, the super family consisted of 74 families wherein each family is involved in the transport of a certain substrate. However, among the 74 families, 49 were yet to be characterized andhence termed as Unknown major facilitators (UMFs)[29].Currently, MFS consists of 249,360 sequences spread across 25 families according to PFAM database (version 27.0) description of the superfamily. MFS superfamily proteins play crucial roles in many diseases through the aberrant action of drug transport leading to drug resistance. Often, resistance to antibiotics is correlated with the action of MFS resistance genes[30]. In like manner, mutations within the MFS transporters may lead to several neurodegenerative diseases and vascular disorders of the brain[31-32].BT1 family members are transmembrane proteins that function as Biopterin transporters. There are several putative BT1 proteins in Leishmania that are involved in pteridine transport. The protozoan parasites Leishmaniaare pteridineauxotrophs and hence require an exogenous source[33-34]. Gradually, they have evolved a versatile pteridine salvage network to accumulate and reduce pteridines. This network system includes biopterin transporters (BT1) among other Folate transporters (FT1) and pteridinereductases (PTR1). This salvage network is predominant during the infectious life cycle of Leishmania parasites which helps in its resistance to antifolates. The accumulation of pteridines occurs via two distinct plasma membrane proteins, namely BT1 and FT1[35]. BT1 helps in the Biopterin transport whereas FT1 is actively involved in Folate Transport, and both are essential for the survival of the pathogen[36].We performed phylogenetic analysis of BT1 family to understand the phyletic relationship and distribution of E9BPD7 amongst the members of BT1 family. To eliminate any artefacts arising out of methodology, in the present study we built the ML tree using MEGA (100 bootstrap) and RaXML (500 bootstrap) and a NJ tree using MEGA (500 bootstrap). These 3 trees are comparable with the earlier work shown by Pao et al. (1998) [26] and all the 17-member families cluster identical to the phylogenetic tree previously reported. Each member family is annotated with a different colour in Figure 3A and Figure 3B.

Owing to the finer sequence differences, we would expect BT1 family members to cluster differentially in the presence of members from other MFS families. As previously discussed, to eliminate any artefacts arising out of methodology, ML tree was built using MEGA and RaXML. Figure 4A and 4B shows BT1 protein sequences (coloured in black) clustering as a separate group, clearly indicating that BT1 family is indeed a distinguishable family of MFS. More importantly, our query of interest (E9BPD7) clusters well within the clad of BT1 family strongly suggesting that E9BPD7 is a member of BT1 family.Though Trypanosomatids belong to the Eukaryotic domain, there are numerous differences between these parasitic protozoans and other higher order eukaryotic species. These differences could also be reflected within the protein domains shared by these pathogens with other eukaryotic species. It is apparent from the tree shown in Figure 5A (MEGA) and Figure 5B (RaXML) that BT1 sequences from Trypanosomatids form a distinctive cluster (coloured RED) while other eukaryotic sequences can be categorized into 3 different subfamilies based on phylogenetic clustering. However, our sequence of interest in this case study, E9BPD7 (in BLACK), interestingly clusters with sequences from higher organisms (coloured BLUE). This suggests that our query of interest, E9BPD7 contains sequence characteristics distinct from other BT1 sequences within the phyla of Trypanosomatids.

To further explore the key sequence features and motifs of each of these sub-clusters, we performed sequence based motif analysis. From Figure 6, it is evident that the sequence motifs from clusters coloured in Blue (Figure 6A), Purple (Figure 6B) and Green (Figure 6C) are very similar. The conserved sequence signature shared by the individual motifs from the three clusters is preserved within the combined sequence motif that was generated using all the sequences from the Blue, Purple and Green clusters (Figure 6D). However, the motif belonging to the Red cluster (Figure 6E) with sequences from Trypanosomatids that contain a BT1 domain is unique in nature. Therefore, based on the sequence motif and conserved signatures, BT1 family can further be classified into two different subfamilies - a subfamily exclusively containing sequences from Trypanosomatid phyla that share a unique sequence motif and another family containing non-trypanosome sequences. It is also interesting to note that besides the classical members, trypanosomatids also contain members like E9BPD7 which share a non Trypanosomatid like BT1 motif.The sequence motif/signature information for BT1 family (Trypanosomatids and non-Trypanosomatids) that has been proposed in the present study may help in further mining and characterization of this important family of transporters.

## Conclusion

The application of various computer aided prediction tools has enabled us to characterise and assign putative functional information to 36 hypothetical membrane proteins in Leishmaniadonovani.This information could potentially aid in identifying drug targets for the treatment and cure of VL in the future. These 36 sequences have been annotated with functional information from PFAM and GO. Such crucial data may prove to be of great assistance in deciphering the biological roles and molecular functions of these hypothetical proteins in the protozoan. Based onour observation, it can be noted that,35 of these 36 proteins have been associated to a COG family as well. A thorough sequence characterization of E9BPD7, an uncharacterised hypothetical protein inLeishmaniadonovani which does not find an association in COG database, has been undertaken. Using phylogenetic studies, E9BPD7 has been proposed to be a member of the BT1 family of MFS with high confidence. The importance of this protein belonging to MFS superfamily is obvious, as its roles are imminent in many diseases,and drug transport mechanism. Often, the resistance to antibiotics is correlated with the action of MFS resistance genes [30].Mutations within the MFS transporters may lead to several neurodegenerative diseases and vascular disorders of the brain as well[31-32].Further, the analysis with E9BPD7 revealed interesting information about the presence of a non-Trypanosomatid sequence motif in this sub-family. Additionally, two different sub-families, viz., Trypanosomatids sub-family and non-Trypanosomatids subfamily have been proposed within the BT1 family, based on phyletic clustering and presence of class specific sequence motifs.These two explicit motif signatures would be of immense help in sequence mining and characterization of these important classes of membrane proteins in Trypanosomatids and other organisms. Many such novel sequences within Trypanosomatids, appear to play crucial roles in the biology of these pathogens. 

## Figures and Tables

**Table 1:** GO terms, PFAM domain associations, homologs picked during the BLAST step in the BLAST2GO analysis of the 36 sequences.

Sequence name	Seq. Length	#Hits	min. eValue	mean Similarity	#GOs	GOs	PFAM	COG
tr|E9BRP4|E9BRP4_LEIDB	448	12	3.64E-27	49.33%	11	C:plasmodesma; P:vacuole organization; C:extracellular region; C:membrane; C:plasma membrane; C:cytoplasmic membrane-bounded vesicle; C:integral component of membrane; C:lysosomal membrane; C:lysosome; F:cobalamin binding; P:transport	PF04791.	NOG05360
tr|E9BNE3|E9BNE3_LEIDB	961	20	0	57.20%	5	C:endoplasmic reticulum membrane; C:integral component of membrane; F:GTPase activity; F:GTP binding; P:GTP catabolic process	PF05879.	NOG02311
tr|E9BRS7|E9BRS7_LEIDB	917	3	1.33E-37	42.33%	6	C:endoplasmic reticulum; P:pollen sperm cell differentiation; C:plasma membrane; P:single fertilization; C:integral component of membrane; C:membrane	PF10699.	NOG40221
tr|E9BIV6|E9BIV6_LEIDB	289	4	3.33E-19	48.25%	2	C:membrane; P:localization	PF01988.	COG1814
tr|E9BUL8|E9BUL8_LEIDB	239	20	2.85E-11	48.55%	2	C:membrane; C:cell part		NOG45674
tr|E9BLF1|E9BLF1_LEIDB	609	12	1.33E-19	42.08%	5	F:molecular_function; P:biological_process; C:cellular_component; C:integral component of membrane; C:membrane	PF04791.	NOG05043
tr|E9BUK6|E9BUK6_LEIDB	156	5	6.35E-11	51.80%	4	C:membrane; C:endoplasmic reticulum part; F:phosphatidylinositol N-acetylglucosaminyltransferase activity; P:GPI anchor biosynthetic process	PF08510.	NOG108269
tr|E9BHM4|E9BHM4_LEIDB	480	11	2.98E-51	48.82%	1	C:membrane	PF04791.	NOG05360
tr|E9BPF5|E9BPF5_LEIDB	309	20	2.80E-50	45.85%	4	C:endoplasmic reticulum-Golgi intermediate compartment; C:membrane; C:organelle part; P:vesicle-mediated transport	PF07970.	NOG01122
tr|E9BN53|E9BN53_LEIDB	467	20	9.62E-60	47.25%	6	C:endoplasmic reticulum; C:Golgi apparatus; C:integral component of membrane; C:intracellular organelle part; C:bounding membrane of organelle; P:transport	PF07970.	NOG01122
tr|E9BSS4|E9BSS4_LEIDB	236	20	2.81E-24	47.20%	1	P:transmembrane transport	PF00153.	NOG09792
tr|E9B799|E9B799_LEIDB	97	11	2.57E-22	55.91%	5	C:mitochondrial inner membrane; C:integral component of membrane; F:pyruvate transmembrane transporter activity; P:pyruvate metabolic process; P:mitochondrial pyruvate transport	PF03650.	NOG62075
tr|E9BUT1|E9BUT1_LEIDB	363	20	2.97E-36	46.30%	13	C:endomembrane system; C:integral component of membrane; C:intracellular membrane-bounded organelle; C:cytoplasmic part; F:receptor activity; F:protein binding; F:hormone binding; P:negative regulation of protein phosphorylation; P:fatty acid metabolic process; P:hormone-mediated signaling pathway; P:regulation of signal transduction; P:cytokine-mediated signaling pathway; P:multicellular organismal process	PF03006.	COG1272
tr|E9B8H1|E9B8H1_LEIDB	163	7	1.13E-24	54.57%	3	C:integral component of membrane; C:extracellular vesicular exosome; P:vesicle-mediated transport	PF04178.	COG5102
tr|E9BTD2|E9BTD2_LEIDB	463	5	9.46E-32	43.60%	3	C:integral component of membrane; C:intracellular membrane-bounded organelle; C:cytoplasmic part	PF00892.	COG0697
tr|E9BUT3|E9BUT3_LEIDB	337	20	7.97E-34	51.40%	14	C:Golgi apparatus; C:integral component of membrane; F:receptor activity; F:protein binding; F:hormone binding; P:negative regulation of protein phosphorylation; P:fatty acid metabolic process; P:response to salt stress; P:response to sucrose; P:hormone-mediated signaling pathway; P:cytokine-mediated signaling pathway; P:system development; P:regulation of cellular component organization; P:regulation of intracellular signal transduction	PF03006.	COG1272
tr|E9B7Q6|E9B7Q6_LEIDB	303	10	7.37E-48	53.20%	5	C:plasma membrane; C:integral component of membrane; F:acetate:proton symporter activity; P:acetate transmembrane transport; P:succinate transmembrane transport	PF01184.	COG1584
tr|E9BQC0|E9BQC0_LEIDB	215	20	2.92E-15	49.70%	15	C:endosome; C:endoplasmic reticulum membrane; C:integral component of membrane; P:cell morphogenesis; P:response to unfolded protein; P:macromolecule biosynthetic process; P:response to light stimulus; P:proteasomal protein catabolic process; P:cell growth; P:response to endoplasmic reticulum stress; P:single-organism carbohydrate metabolic process; P:positive regulation of cellular process; P:root hair cell differentiation; P:developmental growth involved in morphogenesis; P:single-organism intracellular transport	PF04511.	COG5291
tr|E9BNF8|E9BNF8_LEIDB	363	20	7.94E-26	42.00%	13	C:endomembrane system; C:membrane; C:intracellular membrane-bounded organelle; C:cytoplasmic part; P:lipid transport; P:organic anion transport; P:cellular component organization; P:cell differentiation; P:multicellular organismal process; P:anatomical structure development; P:regulation of cellular process; P:response to stimulus; P:cellular localization	PF03381.	COG5035
								
tr|E9BBZ8|E9BBZ8_LEIDB	230	7	6.24E-17	51.14%	4	C:endoplasmic reticulum; C:membrane; P:single-organism carbohydrate metabolic process; P:organic substance biosynthetic process	PF04193.	NOG29600
								
tr|E9BKX1|E9BKX1_LEIDB	681	20	6.37E-101	49.90%	7	C:endosome; C:vacuolar membrane; C:Golgi apparatus; C:plasma membrane; C:integral component of membrane; P:transport; P:cellular transition metal ion homeostasis	PF02990.	NOG01714
tr|E9BSL6|E9BSL6_LEIDB	421	20	1.32E-48	41.20%	13	C:endomembrane system; C:membrane; C:intracellular membrane-bounded organelle; C:cytoplasmic part; C:intracellular organelle part; P:cellular component organization; P:macromolecule localization; P:single-organism cellular process; P:single-organism transport; P:regulation of cellular process; P:response to stimulus; P:cellular localization; P:organic substance transport	PF03381.	COG5035
tr|E9BE08|E9BE08_LEIDB	253	20	1.44E-23	48.15%	15	C:endoplasmic reticulum membrane; F:protein binding; P:proteolysis; P:response to red or far red light; P:cell tip growth; P:protein transport; P:carbohydrate biosynthetic process; P:cellular response to unfolded protein; P:response to endoplasmic reticulum stress; P:cellular protein metabolic process; P:single-organism transport; P:intracellular transport; P:root hair elongation; P:regulation of cellular process; P:multi-organism process	PF04511.	COG5291
tr|E9BK55|E9BK55_LEIDB	528	6	4.63E-66	49.67%	2	C:integral component of membrane; P:transmembrane transport	PF07690.	COG0477
tr|E9BQF9|E9BQF9_LEIDB	584	11	6.53E-85	56.18%	1	C:integral component of membrane	PF05602.	NOG01500
tr|E9BB63|E9BB63_LEIDB	293	17	2.79E-27	50.35%	9	F:molecular_function; P:positive regulation of synaptic transmission, cholinergic; C:cellular_component; C:endoplasmic reticulum; P:biological_process; C:integral component of membrane; C:membrane; P:nervous system development; P:motor neuron axon guidance		COG0398
tr|E9B7V0|E9B7V0_LEIDB	250	20	6.39E-18	45.50%	3	C:membrane; F:phosphotransferase activity, for other substituted phosphate groups; P:phospholipid biosynthetic process	PF01066.	COG5050
tr|E9BV56|E9BV56_LEIDB	372	20	2.96E-51	50.45%	3	C:endosome; C:membrane; P:regulation of localization	PF03619.	NOG02767
tr|E9B8Z6|E9B8Z6_LEIDB	642	18	8.96E-55	47.50%	1	C:membrane	PF02487.	NOG230622
tr|E9BC38|E9BC38_LEIDB	140	9	2.31E-22	50.11%	2	C:mitochondrial inner membrane; P:mitochondrial pyruvate transport	PF03650.	NOG111897
tr|E9BRM8|E9BRM8_LEIDB	341	20	2.90E-37	49.60%	9	C:endomembrane system; C:integral component of membrane; C:intracellular membrane-bounded organelle; C:cytoplasmic part; F:nucleotide-sugar transmembrane transporter activity; P:nucleotide-sugar transport; P:cellular macromolecule biosynthetic process; P:single-organism cellular process; P:glycosylation	PF03151.	COG5070
tr|E9BM11|E9BM11_LEIDB	325	20	1.82E-48	47.05%	9	C:endomembrane system; C:intracellular membrane-bounded organelle; C:cytoplasmic part; F:pyrimidine nucleotide-sugar transmembrane transporter activity; P:organic anion transport; P:pyrimidine nucleotide-sugar transport; P:primary metabolic process; P:single-organism cellular process; P:organic substance metabolic process	PF03151.	NOG00787
tr|E9BA67|E9BA67_LEIDB	221	20	6.00E-12	53.40%	89	F:molecular_function; P:biological_process; C:cellular_component; F:1-phosphatidylinositol-4-phosphate 5-kinase activity; P:pollen germination; P:endocytosis; C:pollen tube; P:phosphatidylinositol metabolic process; C:apical plasma membrane; P:establishment of tissue polarity; P:pollen tube growth;	PF02493.	COG4642
tr|E9BS03|E9BS03_LEIDB	361	20	3.55E-69	59.50%	14	C:mitochondrial outer membrane; C:peroxisomal membrane; C:postsynaptic membrane; F:protein binding; F:ATP binding; F:ATPase activity; P:positive regulation of receptor internalization; P:nematode larval development; P:ATP catabolic process; P:protein targeting to mitochondrion; P:learning; P:memory; P:body morphogenesis; P:negative regulation of synaptic transmission, glutamatergic	PF00004.	COG0464
tr|E9BL96|E9BL96_LEIDB	425	20	2.06E-30	43.25%	1	F:carboxylic ester hydrolase activity	PF12697.	COG0429
tr|E9BPD7|E9BPD7_LEIDB	475	4	7.37E-09	44.50%	2	C:integral component of membrane; P:transport	PF03092.
